# *XRCC3* Thr241Met polymorphism and ovarian cancer risk: a meta-analysis

**DOI:** 10.1007/s13277-013-1357-z

**Published:** 2013-11-20

**Authors:** Yulan Yan, Hongjie Liang, Ruolin Li, Li Xie, Meng Li, Shan Li, Xue Qin

**Affiliations:** Department of Clinical Laboratory, First Affiliated Hospital of Guangxi Medical University, 6 Shuangyong Road, Nanning, 530021 Guangxi People’s Republic of China

**Keywords:** *XRCC3*, Polymorphism, Ovarian cancer, Meta-analysis

## Abstract

Genetic polymorphism of X-ray repair crosscomplementing group 3 (*XRCC3*) Thr241Met has been implicated to alter the risk of ovarian cancer, but the results are controversial. In order to get a more precise result, a meta-analysis was performed. All eligible studies were identified through an extensive search in PubMed, Excerpta Medica Database (Embase), Chinese National Knowledge Infrastructure database, and Chinese Biomedical Literature Database before August 2013. The association between the *XRCC3* Thr241Met polymorphism and ovarian cancer risk was conducted by odds ratios (ORs) and 95 % confidence intervals (95 % CIs). Finally, a total of four publications including seven studies with 3,635 cases and 5,473 controls were included in our meta-analysis. Overall, there was no association between *XRCC3* Thr241Met polymorphism and risk of ovarian cancer under all five genetic models in overall population (T vs. C: OR = 0.99, 95 % CI = 0.960–1.03, *P* = 0.752; TT vs. CC: OR = 1.00, 95 % CI = 0.91–1.10, *P* = 0.943; TC vs. TT: OR = 0.97, 95 % CI = 0.92–1.04, *P* = 0.396, Fig. 1; TT vs. TC/CC: OR = 1.00, 95 % CI = 0.91–1.12, *P* = 0.874; TT/TC vs. CC: OR = 0.98, 95 % CI = 0.94–1.03, *P* = 0.486). In the subgroup analysis according to ethnicity, the results suggested that *XRCC3* Thr241Met polymorphism was not associated with the risk of ovarian cancer in Caucasians population. No significant association was found between the *XRCC3* Thr241 Met polymorphism and the risk of ovarian cancer. Given the limited sample size and ethnicities included in the meta-analysis, further large scaled and well-designed studies are needed to confirm our results.

## Introduction

Ovarian cancer is the eighth most commonly diagnosed cancer and the fifth leading cause of cancer death in the females worldwide with an estimated 225,500 new cases and 140,200 deaths every year [1, 2]. Though there are many advances in the classification, diagnosis, and treatment of ovarian cancer, it is still difficult to diagnose early and causes serious damage to public health [3, 4]. Since the low rate of early detection, the late clinical manifestation, and the lack of effective treatments, the 5-year survival rate for ovarian cancer patients is only about 40 % [1]. What is worse, the mechanism of ovarian carcinogenesis is not unclear yet, and multiple factors may increase the risk of developing ovarian cancer, such as age, gravidity, tubal ligation, number of ovulatory cycles, family history of ovarian cancer as well as lifestyle factors [3, 5]. However, not all of those who expose to those risk factors develop ovarian cancer, which suggests genetic factors may also play an important role in the host’s susceptibility to ovarian cancer [6]. Candidate genes include the insulin receptor substrate 1 [7], lysyl oxidase G473A [8], vitamin D receptor [9], and *MTHFR* C677T [2].

Another candidate gene that has received a lot of attention is the X-ray repair cross-complementing group 3(*XRCC3*). *XRCC3*, located at chromosome 14q32.3, is a member of DNA repair genes and involved in maintaining the stability of genome by homologous recombination repair for DNA double-strand breaks [10, 11]. The Thr241Met is the most common polymorphism of *XRCC3*, which substitutes at codon 241 in exon 7, with a C to T transition (*XRCC3*-18067C/T, rs861539) [11, 12]. We called this SNP in the *XRCC3* gene “-18067C/T” for short in this meta-analysis. Previous studies were performed to investigate the relationship between the *XRCC3*-18067C/T polymorphism and ovarian cancer risk [13–16]. However, the results were controversial, which might be caused by the limitation of individual studies. To assess the association between *XRCC3*-18067C/T polymorphism and ovarian cancer risk more precisely, we conducted a meta-analysis.

## Materials and methods

### Search strategy

To identify all currently available studies on the association between *XRCC3*-18067C/T polymorphism and ovarian cancer risk, we conducted an extensive search in PubMed, Excerpta Medica Database (Embase), Chinese National Knowledge Infrastructure database, and Chinese Biomedical Literature Database before August 2013 by using the terms as follows: “*XRCC3*” or “X-ray cross complementing group 3” in combination with “polymorphism,” “polymorphisms,” “variant,” or “mutation” in combination with “ovarian cancer,” “ovarian carcinoma,” or “ovarian tumor” for all publications. There was no language limitation. Additional studies were identified by a hand search of the references of original studies. Review articles were also examined to find more eligible studies.

### Inclusion and exclusion criteria

Studies to be included in the meta-analysis were required to meet the following criteria: (a) it assessed the association between the *XRCC3*-18067C/T polymorphism and ovarian cancer, (b) a case–control design, (c) the genotype distribution among the control population was consistent with Hardy–Weinberg Equilibrium (HWE), and (d) sufficient reported genotypic frequencies in both cases and controls for estimating an odds ratio (OR) with its 95 % confidence interval (95 % CI). The following exclusion criteria were used for excluding studies: (a) control population including patients, (b) studies contained duplicate data, and (c) case reports or reviews.

### Data extraction

Information was carefully extracted from all eligible publications by two authors independently according to the inclusion criteria listed above, disagreements were resolved through consensus, if could not reached agreement, another author was consulted to resolve the debate. The following information was extracted: the name of first author, year of publication, country of origin, ethnicity of the population, genotyping methods, source of the control group, sample size of cases. Different ethnicities were categorized as Caucasians and mixed.

### Statistical analysis

The possible association between the *XRCC3*-18067C/T polymorphism and ovarian cancer susceptibility was measured by the pooled ORs with corresponding 95 % CIs in five genetic models: allele contrast (T vs. C), homozygote (TT vs. CC), heterozygote (TC vs. CC), recessive (TT vs. TC/ CC), and dominant (TT/TC vs. CC) models, respectively. Heterogeneity was checked by the Chi-square based Q statistic. If the result of the heterogeneity test *P* < 0.10, suggested the between-study heterogeneity was existed, ORs were pooled by random-effects model (DerSimonian and Laird method) [17]. Otherwise, the fixed-effects model (the Mantel-Haenszel method) was used [18]. In addition, the effect of heterogeneity was quantified also by using *I* ^2^ value [19]. If obvious heterogeneity existed (*I* ^2^value >50 % or *P* < 0.10), the overall estimate of risk was calculated by the random-effects model; when obvious heterogeneity was absent, the fixed-effects model was used.

The HWE was assessed by using a professional web-based program (http://ihg2.helmholtz-muenchen.de/cgibin/hw/hwa1.pl) to confirm whether the genotype distribution of XRCC3-18067C/T in controls agreed with HWE if *P* > 0.05 suggests the controls followed HWE balance. Egger’s linear regression test was applied to examine the possible publication bias, Egger’s test (*P* < 0.05 was considered representative of statistically significant publication bias) [20]. Publication bias was also tested by visual observation of funnel plot [21]. Statistical analysis was undertaken using the program STATA Software (version 9.2, Stata Corp). Two-sided *P* < 0.05 was considered statistically significant.

## Results

### Study characteristics

After carefully selecting based on the search criteria above, a total of four publications including seven studies with 3,635 cases and 5,473 controls were included in our meta-analysis [13–16], all of these publications were written in English. There were six studies of Caucasians and one mixed. The genotype distribution in the controls of all the studies included in the meta-analysis were consistent with HWE (all *P* > 0.05). The study characteristics included in the meta-analysis were listed in Table 1.

**Table 1:** General characteristics of studies included in the meta-analysis

First author	Year	Country	Ethnicity	Method of genotyping	Source of control	Sample size (case control)	HWE of control
Auranen(a)	2005	UK	Caucasian	TaqMan	PB	1139/1614	0.395
Auranen(b)	2005	USA	Caucasian	TaqMan	PB	270/344	0.111
Auranen(c)	2005	Danish	Caucasian	TaqMan	PB	361/891	0.080
Hormazabal	2012	Chile	Mix	TaqMan	PB	87/570	0.172
Beesley(a)	2007	Australia	Caucasian	PCR-RFLP	PB	504/972	0.326
Beesley(b)	2007	Australia	Caucasian	PCR-RFLP	PB	731/747	0.950
Webb	2005	Australia	Caucasian	PCR	HB	543/335	0.420

*PCR*-*RFLP* PCR–restriction fragment length polymorphism, *HWE* Hardy–Weinberg equilibrium, *HB* hospital based, *PB* population based

### Quantitative synthesis of data

**Fig. 1:**
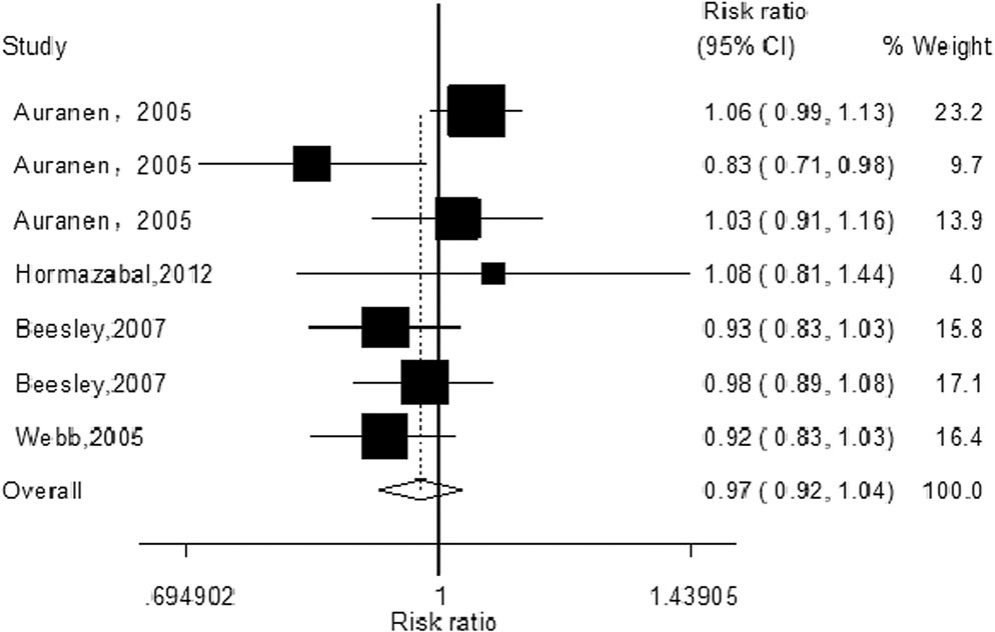
The forest plot describing the meta-analysis under heterozygous model for the association between *XRCC3* Thr241 Met polymorphism and the risk of ovarian cancer in overall population (TC vs. CC)

**Fig. 2:**
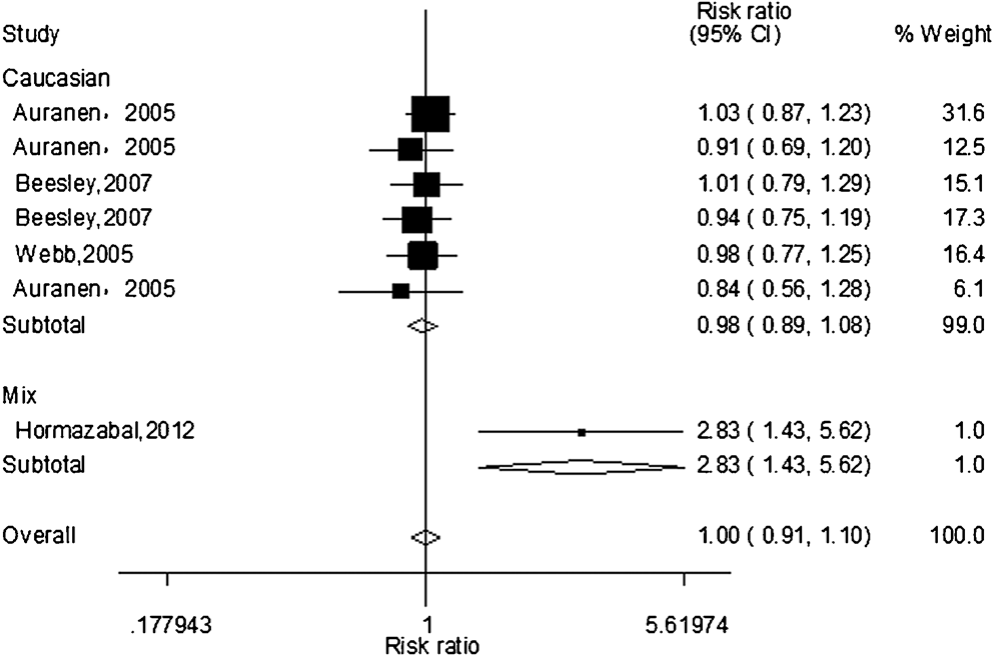
The forest plot describing the meta-analysis under homozygous model for the association between *XRCC3* Thr241 Met polymorphism and the risk of ovarian cancer in Caucasians (TT vs. CC)

The main results of the meta-analysis were listed in Table 2. Meta-analysis of the total studies showed that there was no association between *XRCC3* Thr241Met polymorphism and risk of ovarian cancer under all five genetic models in overall population (T vs. C: OR = 0.99, 95 % CI = 0.96–1.03, *P* = 0.752; TT vs. CC: OR = 1.00, 95 % CI = 0.91–1.10, *P* = 0.943; TC vs. TT: OR = 0.97, 95 % CI = 0.92–1.04, *P* = 0.396, Fig. 1; TT vs. TC/CC: OR = 1.00, 95 % CI = 0.91–1.12, *P* = 0.874; TT/TC vs. CC: OR = 0.98, 95 % CI = 0.94–1.03, *P* = 0.486). In the subgroup analysis according to ethnicity, the results suggested that *XRCC3* Thr241Met polymorphism was not associated with the risk of ovarian cancer in Caucasians population (only shown homozygote model in Fig. 2). Detailed data are shown in Table 2.

**Table 2:** Results of meta-analysis for *XRCC3* Thr241Met polymorphism and ovarian cancer risk

Comparison	Population	*N*	Test of association			Model	Test of heterogeneity	
			OR	95 % CI	*P* value		*P* value	*I* ^2^
T vs. C	Overall	7	0.99	0.96–1.03	0.752	F	0.156	35.6
	Mix	1	1.33	1.03–1.71	0.027	F	–	–
	Caucasians	6	1.00	0.96–1.03	0.507	F	0.534	0
TT vs. CC	Overall	7	1.00	0.91–1.10	0.943	F	0.111	42.0
	Mix	1	2.83	1.43–4.91	5.62	F	–	–
	Caucasians	6	0.98	0.89–1.08	0.658	F	0.933	0
TC vs. CC	Overall	7	0.97	0.92–1.04	0.396	R	0.068	48.8
	Mix	1	1.08	0.81–1.44	0.590	F	–	–
	Caucasians	6	0.97	0.91–1.03	0.336	R	0.045	55.9
TT vs. TC/CC	Overall	7	1.00	0.91–1.12	0.874	F	0.131	39.1
	Mix	1	2.83	1.40–5.75	0.004	F	–	–
	Caucasians	6	0.99	0.89–1.10	0.852	F	0.908	0
TT/TC vs. CC	Overall	7	0.98	0.94–1.03	0.486	R	0.083	46.3
	Mix	1	0.99	0.96–1.02	0.175	F	–	–
	Caucasians	6	0.99	0.95–1.02	0.426	F	0.104	45.2

*OR* odds ratio, *CI* confidence interval, *F* fixed-effects model, *R* random-effects model

### Heterogeneity analysis and publication bias analysis

There was a significant heterogeneity found under heterozygote (TC vs. CC) and dominant (TT/TC vs. CC) genetic models in overall population. To examine the source of heterogeneity, we assessed the dominant model (TT/TC vs. CC) by ethnicity. However, the results showed there was no obvious heterogeneity in the subgroup analyses of both Caucasians and mix, suggesting that ethnicity was the major source of heterogeneity in our meta-analysis.

**Fig. 3:**
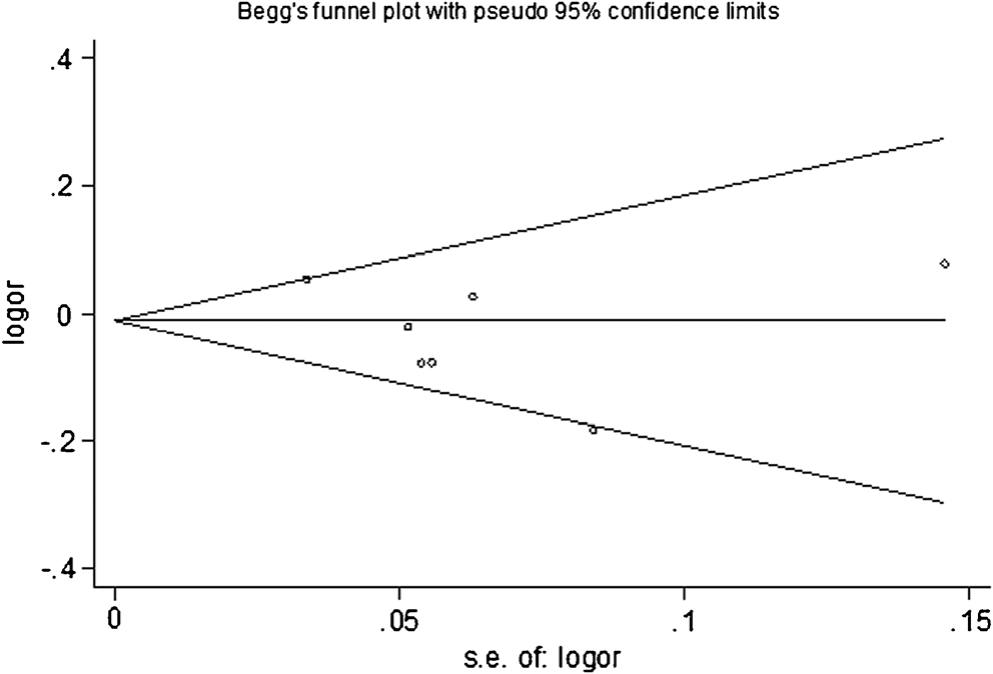
Begg funnel plot for publication bias test for the association between *XRCC3* Thr241 Met polymorphism and the risk of ovarian cancer under heterozygous model (TC vs. CC). Each point represents a separate study for the indicated association. *Log [OR]* natural logarithm of OR. *Horizontal line* means effect size

Funnel plot and Egger’s test were used to assess the publication bias in this meta-analysis. Funnel plot is relatively straightforward to observe whether the presence of publication bias and Egger’s test was provided statistical evidence of symmetries of the plots. As a result, both the shape of the funnel plot and Egger’s test value did not suggest any evidence of obvious asymmetry (Fig. 3, all *P* > 0.05, data not shown).

## Discussion

It is well known that SNPs are the most common sources of human genetic variation, and they may contribute to an individual’s susceptibility to cancer. *XRCC3* plays a critical role in maintaining genomic integrity by repairing radiation-induced DNA double-strand breaks, and the *XRCC3* polymorphism may affect the DNA repair capacity of its encoded protein, thus, contributes to the development of cancers [11, 12].

Since the identification of *XRCC3* Thr241Met polymorphism, a growing number of studies suggested that *XRCC3* Thr241Met polymorphism plays an important role in the development of cancers, such as glioma [22], hepatocellular carcinoma [23], head and neck cancer [24], lung cancer [25], and so on. Many published studies that aim at the role of *XRCC3* Thr241Met polymorphism in ovarian cancer risk have been performed, but the results are controversial. Different study designs, various methodologies, and different population backgrounds would inevitably increase the risk, which should be responsible for the controversial conclusions. However, meta-analysis is a powerful tool for analyzing cumulative data of studies which the individual sample sizes are small and the statistical power is low. No meta-analysis evaluating on the association between the *XRCC3* Thr241Met polymorphisms and ovarian cancer risk has been performed. So, we carried out a meta-analysis.

A total of four publications including seven studies with 3,635 cases and 5,473 controls were included in our meta-analysis [13–16]. The results of the meta-analysis showed that *XRCC3* Thr241Met polymorphism was not associated with ovarian cancer risk in the overall populations. Subgroup analysis based on ethnicity indicated that *XRCC3* Thr241Met polymorphism was not a risk factor for Caucasians, but is a risk for mix. The results about mix may not be accurate because only one study was found about mix; the sample size is too small to explain the conclusion.

The heterogeneity is a very important part of a meta-analysis, and finding the possible sources for the high heterogeneity is very important [26, 27]. The studies included in our meta-analysis probably have different genetic backgrounds, environmental exposures, methodology, and sample size, thus, caused the inconsistent conclusions. In this study, obvious heterogeneity between-study was found under heterozygote and dominant genetic models in the overall population. To explore the possible sources for the heterogeneity in the meta-analysis, we performed subgroup analysis by ethnicity. Through subgroup analysis, we found that ethnicity was the major source of the heterogeneity in our meta-analysis, which could be explained by the race-specific effect of *XRCC3* Thr241Met polymorphism and the susceptibility of ovarian cancer that is because different countries may have different genetic backgrounds and environmental exposures. Publication bias is another important factor which may have a negative effect on the meta-analysis. Both Funnel plot and Egger’s test were used to assess the publication bias of our meta-analysis. The shape of funnel plot and statistical results show no obvious publication bias found; this indicates that the publication bias has little effect on the results of our study, and the results of our meta-analysis are relatively stable.

There are several limitations in our meta-analysis that should be considered. Firstly, the sample sizes are relatively small; there are only seven studies with a total of 3,635 cases and 5,473 controls included in our meta-analysis. More studies with large sample size and well-designed are needed to further identify this association more comprehensively. Secondly, studies included in the present meta-analysis mainly provided data towards Caucasians in the light of the race-specific association probably exist; other ethnicities including Asians, Africans, and others should be investigated in future studies. Thirdly, subgroup analyses according to age, radiation exposure, histological types, and other elements have not been performed due to insufficient relevant data available in the primary studies. Finally, only published and English studies were included in this study; thus, publication and potential language biases may occur.

In summary, our meta-analysis systematically analyzed the association between *XRCC3* Thr241Met polymorphism and the risk of ovarian cancer. No significant association was found between the *XRCC3* Thr241 Met polymorphism and the risk of ovarian cancer. Given the limited sample size and ethnicities included in the meta-analysis, further larger scaled and well-designed studies are needed to confirm our results.
